# Evaluation of Obstructive Sleep Apnea Phenotypes Treatment Effectiveness

**DOI:** 10.3390/medicina57040335

**Published:** 2021-04-01

**Authors:** Karolina Charčiūnaitė, Rasa Gauronskaitė, Goda Šlekytė, Edvardas Danila, Rolandas Zablockis

**Affiliations:** 1 Faculty of Medicine, Vilnius University, 03101 Vilnius, Lithuania; 2 Clinic of Chest Diseases, Immunology and Allergology, Institute of Clinical Medicine, Vilnius University, 03101 Vilnius, Lithuania; rasa.gauronskaite@santa.lt (R.G.); edvardas.danila@santa.lt (E.D.); rolandas.zablockis@santa.lt (R.Z.); 3 Centre of Pulmonology and Allergology, Vilnius University Hospital Santaros Klinikos, Santariskiu st. 2, 08661 Vilnius, Lithuania; goda.slekyte@santa.lt

**Keywords:** obstructive sleep apnea, phenotype, cluster, CPAP, treatment effectiveness

## Abstract

*Background and Objective*: Obstructive sleep apnea (OSA) is a heterogeneous chronic sleep associated disorder. A common apnea-hypopnea index (AHI)-focused approach to OSA severity evaluation is not sufficient enough to capture the extent of OSA related risks, it limits our understanding of disease pathogenesis and may contribute to a modest response to conventional treatment. In order to resolve the heterogeneity issue, OSA patients can be divided into more homogenous therapeutically and prognostically significant groups–phenotypes. An improved understanding of OSA phenotype relationship to treatment effectiveness is required. Thus, in this study several clinical OSA phenotypes are identified and compared by their treatment effectiveness. *Methods and materials*: Retrospective data analysis of 233 adult patients with OSA treated with continuous positive airway pressure (CPAP) was performed. Statistical analysis of data relating to demographic and anthropometric characteristics, symptoms, arterial blood gas test results, polysomnografic and respiratory polygraphic tests and treatment, treatment results was performed. *Results*: 3 phenotypes have been identified: “Position dependent (supine) OSA” (Positional OSA), “Severe OSA in obese patients” (Severe OSA) and “OSA and periodic limb movements (PLM)” (OSA and PLM). The highest count of responders to treatment with CPAP was in the OSA and PLM phenotype, followed by the Positional OSA phenotype. Treatment with CPAP, despite the highest mean pressure administered was the least effective among Severe OSA phenotype. *Conclusions*: Different OSA phenotypes vary significantly and lead to differences in response to treatment. Thus, treatment effectiveness depends on OSA phenotypes and treatment techniques other than CPAP may be needed. This emphasizes the importance of a more individualized approach when treating OSA.

## 1. Introduction

Obstructive sleep apnea (OSA) is a common chronic sleep associated disorder, characterized by recurring airway obstruction and causing oxygen desaturation, sleep fragmentation and excessive daytime sleepiness [1]. Morbidity rate of OSA in the common population is between 9 to 38% and is currently hire among males, older and overweight people [2]. Although OSA is often asymptomatic and unworthily underrated, it is associated with a higher risk of developing secondary arterial hypertension, atrial fibrillation, stroke, coronary artery disease [3], heart failure [4], diabetes [5], vehicle accidents, deterioration of cognitive function and overall lower quality of life [1,6]. Loud snoring, obesity, increased circumference of the neck as well as an elevation in Epworth sleepiness scale scores allow doctors to suspect the disorder, but polysomnography still remains as the standard of OSA diagnostics [6].

Besides the commonly mentioned cardiovascular risks, OSA can be related to other comorbidities. Recent studies have shown OSA patients having higher prevalence of psoriasis compared to general public [7]. Another study found a high co-existence of OSA in patients with bronchial asthma and an up to 95% prevalence in severe asthma, exacerbating symptoms and deteriorating asthma outcomes [8]. There is also an association between OSA and hypothyroidism found, although evidence is inconclusive [9]. A high number of comorbidities can burden the disease, thus broader evaluation and multifactorial treatment of OSA patients is required.

So far there is no universal symptom, indicator or measurement found that would take into account the polymorphism of the disorder and at the same time accurately evaluate its related risks as well as the impact on disease outcomes. An AHI-focused approach to OSA severity evaluation may contribute negatively to a limited understanding of the disease and lower than expected effect of treatment with continuous positive airway pressure (CPAP) [10]. In the aim to resolve this issue of heterogeneity, we can classify patients into smaller, more homogenous groups, thus, so called phenotypes can be identified. Phenotypes are clinically significant categories of patients, that differ among each other by one or few features and that have the impact on development of symptoms, treatment effectiveness, quality of life and disease outcomes [10]. As prior research shows, phenotyping can aid in further understanding of the disease pathogenesis [11,12], predicting response to treatment [13,14] and evaluating risk of adverse events [15].

A few OSA phenotypes have been recognized in previous studies and are mostly distinguished by the sleep disordered breathing related to or independent of stage of sleep or sleeping position [10,12]. However, there is still a shortage of research, studying the subject of different OSA phenotypes relationship with disease outcomes, prognosis, and an improved understanding of OSA phenotype correspondence to treatment effectiveness is required. Thus, the aim of this study is to identify and compare obstructive sleep apnea clinical phenotypes and find differences in their treatment efficacy.

## 2. Materials and Methods

### 2.1. Study Population

Retrospective data analysis of adult patients with obstructive sleep apnea (OSA), hospitalized in Centre of Pulmonology and Allergology, Vilnius University Hospital Santaros Klinikos between the year of 2014 and 2019, has been performed. Depersonalized data from the hospital database have been received. During the hospitalization every patient has signed an informed consent, allowing the usage of depersonalized data for retrospective studies. The study was approved by the Vilnius Regional Biomedical Ethics Committee (no. 158200-13-652-210) on 3rd of July, 2013, Vilnius, Lithuania.

### 2.2. Data Collection

Inclusion criteria: obstructive sleep apnea diagnosed according to ICSD-3 (International Classification of Sleep Disorders-3) criteria [16], for diagnostics a polysomnography must have been performed, OSA treated with either fixed pressure CPAP, automatic continuous positive pressure CPAP (autoCPAP) or two level positive pressure (BiLevel) devices. Exclusion criteria included: polysomnography has not been performed, primary diagnosis stated chronic obstructive pulmonary disease, mixed or central sleep apnea, refusal of CPAP treatment, lack of polysomnographic data found in the database.

**Figure 1:**
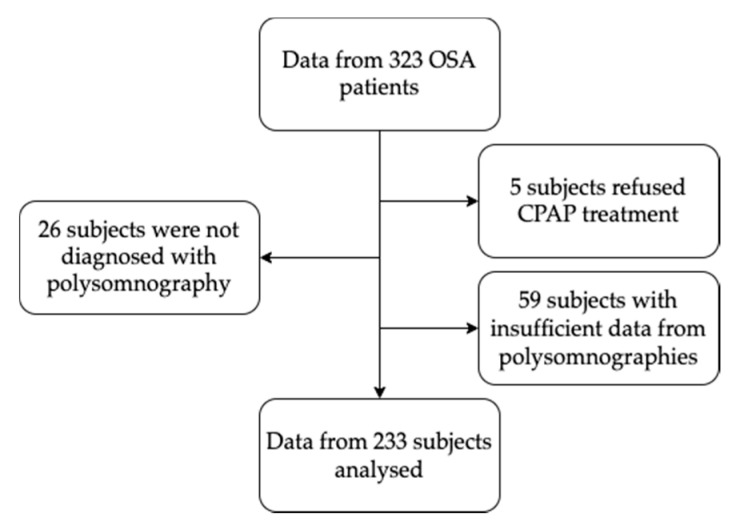
Selection of study subjects. Abbreviation: CPAP - continuous positive airway pressure, OSA–obstructive sleep apnea.

In this study 233 patients’ data has been analyzed. The selection of patients is graphically shown in the Figure 1.

Data relating to demographic, anthropometric characteristics, symptoms, arterial blood gas test results, polysomnografic and respiratory polygraphic test data were obtained from the medical records. Performed treatment as well as adherence to treatment has been analyzed.

CPAP pressure (PEEP) was titrated with an autoCPAP device [17].

### 2.3. Statistical Analysis

Descriptive statistics has been performed, for quantitative variables means, medians, standard deviations and confidence intervals have been counted. For the dispersity of data Skewness and Kurtosis have been performed. For variable comparison Student *t*-test criterion for independent samples and Mann-Whitney U test criterion have been used. Normally distributed data in the results is reported as mean and standard deviation, non-normally distributed data–as median and interquartile range (LQ-UQ). P value less than 0.05 considered significant.

Hierarchical analysis and K-mean cluster methods were used to identify phenotypes. Hierarchical analysis was performed using Ward linkage method.

Statistical analysis was performed using the Statistical Package for Social Science software version 25.0 for Windows (IBM Corp., Armonk, NY, USA).

## 3. Results

### 3.1. Characteristics of the Study Population

Analysis of 233 patients’ anthropometric, clinical, arterial blood gas test and polysomnographic data has been performed. Characteristics of the study population are summarized in Table 1. Polysomnographic data are summarized in Table 2.

64.4% of patients received treatment with fixed pressure CPAP devices, 28.8%-continuous positive pressure CPAP devices (autoPAP) and 6.9% were treated with two level positive pressure devices (BiLevel). 84.9% of study population chose full face masks and the remaining 15.1%-nasal masks. Response to treatment was measured by 3 levels according to AHI differences before and after treatment (ΔAHI): ΔAHI < 5 times/hour has been reached by 53.1% of patients, ΔAHI < 10 times/hour or AHI decrease by 50% was seen in 79.5% and AHI decrease by 50% was reached in as much as 97.8% of patients. Epworth sleepiness scale scores after treatment decreased by almost a half to 5.6 ± 4.0.

### 3.2. Phenotypes

**Figure 2:**
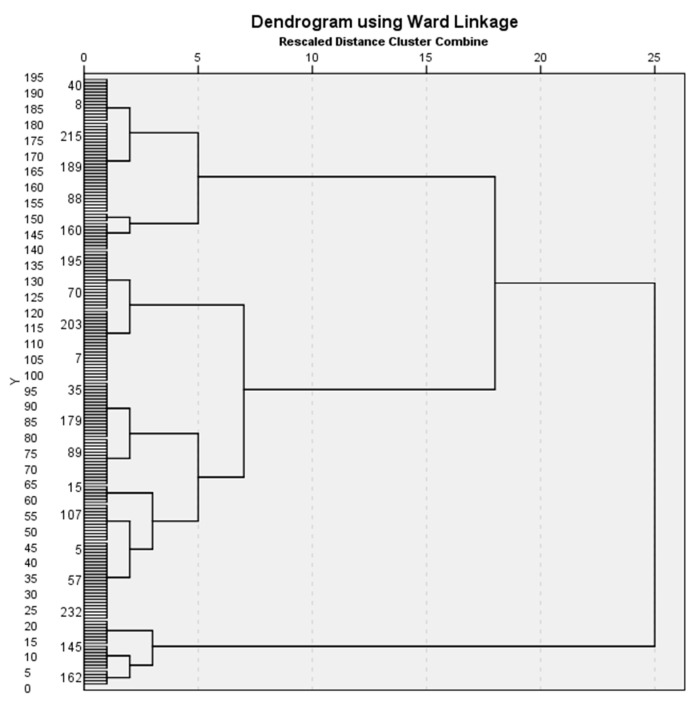
Dendrogram, which indicates 3 distinct main clusters.

9 variables with the most data have been selected for hierarchical analysis: body mass index (BMI), systolic arterial blood pressure, daytime saturation of oxygen in the arterial blood (SaO_2_), partial carbon dioxide pressure in arterial blood (PaCO_2_), partial oxygen pressure in arterial blood (PaO_2_), apnea-hypopnea index (AHI), periodic limb movement index (PLMS), supine AHI, non-supine AHI. These easily measurable variables are important when evaluating disease severity and were chosen in the means to search for more universal (broader) phenotypes that would include as much of the sample size in the analysis as possible. Data concerning different stages of sleep was omitted and not included in the analysis due to data collecting inconsistencies in the database. By performing hierarchical analysis, 3 clusters have been identified. Dendrogram, according which clusters were identified is shown in Figure 2. By performing K-mean cluster analysis, 194 subjects were categorized into 3 statistically different clusters or phenotypes: 1st cluster–“Position dependent (supine) OSA” (further-Positional OSA), 2nd cluster–“Severe OSA in obese patients” (further-Severe OSA) and 3rd cluster-“OSA and periodic limb movements (PLM)” (further-OSA and PLM). 39 subjects were not assigned to any of the clusters. Characteristics of different clusters are summarized in Table 3.

#### 3.2.1. Positional OSA

This is the largest cluster involving 106 patients. The distinctive feature of this phenotype is a higher supine AHI (48.0 times/hour), compared to lower total AHI (37.3 times/hour) and non-supine AHI (24.8 times/hour). Subjects with this phenotype had the lowest: BMI (37.6 kg/m^2^), waist circumference (122.8 cm), AHI, arousal index (ARI) (62.3 times/hour), oxygen desaturation index (ODI) (31.2 times/hour), non-supine AHI. This phenotype had the highest oxygen concentration in the blood during polysomnography (mean SpO_2_–92.0%, nadir SpO_2_-73%), as well as the highest PaO_2_ (76.0 mmHg) and lowest PaCO_2_ (41.4 mmHg) compared to other phenotypes. Despite that, Epworth sleepiness scale score after treatment for this phenotype remained the highest (6.3 points).

#### 3.2.2. Severe OSA

There are 59 subjects in this cluster. The differential features in this phenotype are a significantly higher BMI (42.1 kg/m^2^) and AHI (74.1 times/hour) scores. The increase in AHI is not dependent on position during sleep (supine AHI–76.0 times/hour, non-supine AHI–71.4 times/hour). Mean systolic arterial blood pressure is observed to be significantly higher than in other phenotypes (139.0 mmHg), as well as poorer arterial blood gas test results are seen (PaCO_2_–43.6 mmHg, PaO_2_–68.9 times/hour). Subjects in this group had the lowest oxygen saturation during polysomnography (mean SpO_2_–88.1%, nadir SpO_2_–63.1%). Compared to other phenotypes, this cluster complained about excessive daytime sleepiness more.

#### 3.2.3. OSA and PLM

This is the smallest cluster with 29 patients. The distinctive feature of this cluster is the significant increase in periodic limb movement index (PLMS)–on average 124.9 times/hour. Compared to other phenotypes, systolic blood pressure was the lowest (130.0 mmHg), as well as the neck circumference (44.1 cm). This phenotype had the highest arousal index (ARI) (92.3 times/hour) and snoring percentage (42.4%). AHI averaged at 45.2 times/hour. Interestingly, among all phenotypes, there were the most currently smoking subjects in this group.

### 3.3. The Effectiveness of Treatment

**Figure 3:**
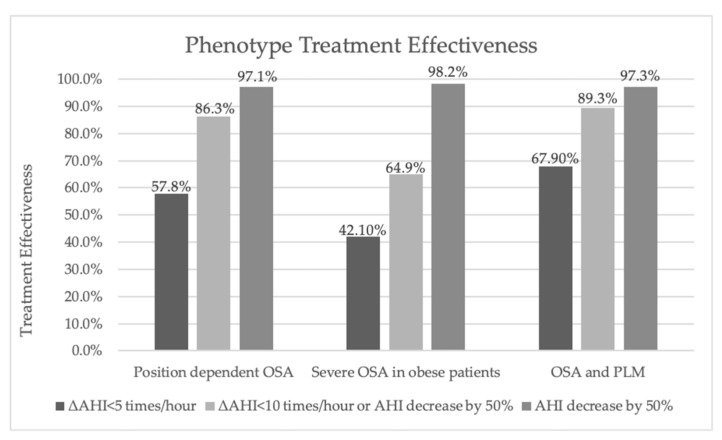
Treatment effectiveness according to phenotypes. Response to treatment is shown in 3 different levels. ΔAHI indicates apnea-hypopnea index rate after treatment with CPAP. Abbreviations: OSA–obstructive sleep apnea, PLM–periodic limb movements, CPAP–continuous positive airway pressure.

Treatment effectiveness was determined by measuring AHI and Epworth sleeping scale scores before and after treatment with CPAP. AHI score after treatment averaged at 5.9 ± 4.8 times/hour (0.3–14.4 times/hour). Epworth sleepiness scale (ESS) score after treatment lowered to 5.6±4.0 points at average (0.0–21.0 points). Treatment when comparing AHI and ESS scores was most effective in OSA and PLM phenotype (ΔAHI–4.6 times/hour, ΔEMS–4.5 points) and least effective according to AHI score in Severe OSA phenotype (ΔAHI–8.6 times/hour). Highest ESS scores after treatment were observed in the Positional OSA phenotype (ΔEMS–6.3 points). Response to treatment when considering AHI scores after treatment and dividing it into 3 categories is compared graphically in the Figure 3. The highest count of treatment responders were in the OSA and PLM phenotype, with the mean of CPAP pressure at 10.0 ± 1.9 cmH_2_O. Second highest count of responders to treatment where in the Positional OSA phenotype with the lowest mean CPAP pressure at 9.39 ± 2.0 cmH_2_O. Despite the highest mean CPAP pressure of 10.9 ± 1.7 cmH_2_O used for treatment, the lowest number of responders was found among Severe OSA phenotype.

## 4. Discussion

The principal indicator of obstructive sleep apnea syndrome severity, treatment effectiveness and prognosis remains apnea-hypopnea index, however, it does not reflect syndrome heterogeneity [10]. Thus, the aim of this study was to analyze other possible methods of syndrome classification, such as phenotyping, consequently contributing to the search of clinically and prognostically relevant patient groups.

Cluster analysis showed 3 main phenotypes: OSA and PLM, Positional OSA and Severe OSA.

Positional OSA was distinguished by the increase in supine AHI value. Positional OSA is generally diagnosed when there is a two-fold increase in AHI compared to non-supine AHI [18], which is also seen in our study. This largest phenotype despite the lowest AHI values, highest oxygen saturation during polysomnography, highest PaO_2_ levels in arterial blood had the least significant change in Epworth scores after treatment with CPAP. Response to treatment for this phenotype was the second highest with the lowest mean CPAP pressure. A greater response to treatment may have been achieved due to these patients lower BMI and lesser disease severity [19]. In recent reports it has been proved, that position therapy in addition to CPAP therapy can increase OSA treatment effectiveness [20].

Severe OSA phenotype had the highest BMI and AHI rates, and was the most symptomatic (according to EDS percentage). In addition, the highest systolic blood pressure measurements, as well as the poorest arterial blood gas test results and lowest oxygen concentrations during polysomnography have been observed compared to other phenotypes. These patients did not display hypercapnia signs and were not diagnosed with obesity hypoventilation syndrome. Treatment with CPAP was the least effective, compared to remaining phenotypes, even though the highest mean pressure was administered. The poorer treatment effect of this phenotype could potentially be burdened by obesity and a higher number of comorbidities, uninvestigated underlying diseases, lesser adherence to treatment, other factors. However, more extensive studies need to be made to investigate and prove these statements.

The distinct feature of OSA and PLM phenotype was the increase in PLMS index value. Periodic limb movement disorder is an often occurring and important sleep related disorder, however, the rise of PLMS index alone, which is in fact often elevated in OSA patients, is not sufficient enough to prove the disorder [21]. These patients also showed higher values of arousal index (ARI), snoring percentage and lower systolic blood pressure. Periodic limb movements can cause awakenings during the night [22]. Frequent arousals during sleep could contribute to lower quality of sleep [23], although our results did not show a significant difference, possibly due to the small sample size of this phenotype. In theory, management of periodic limb movement disorders could potentially improve patients’ quality of sleep, however, due to the lack of evidence, pharmacological therapy is rarely prescribed [21]. Treatment with CPAP was the most effective in this group, compared to remaining phenotypes.

Response to treatment with CPAP of phenotypes with different symptom groups was researched in a recent Icelandic study. In this study, all of the phenotypes responded differently to treatment and the best response to treatment with CPAP was reached with the most symptomatic patients [13]. Another study, comparing the effectiveness of mandibular advancement splints in OSA patients, discovered that supine-predominant patients had lower rates of treatment response, compared to non-positional OSA patients [14].

A similar phenotype to OSA and PLM was found in a Korean study where, similarly to our study, it was the least common and with the highest PLMS count, oldest patient group and lowest Epworth sleepiness scale scores. The main difference to our study, was that these patients had the lowest quality of sleep, which contributes to higher arousal and awakening rates mentioned above [24]. In another recently published study, a phenotype related to periodic limb movements was connected to a two-fold higher cardiovascular or cerebrovascular event risk [25].

A close to Severe OSA phenotype, which displayed AHI average of almost 70 times/hour, was described in the earlier mentioned Korean study, and was associated with a higher cardiovascular and cerebrovascular event risk [24]. Another study compared different phenotypes between men and women and discovered, that both sexes had phenotypes with severe OSA features [26]. One more study researched OSA comorbidities and divided subjects into six clusters of which two clusters were related to severe OSA and obesity, one cluster associated with higher Epworth scores, the highest BMI and severe comorbidities [27], which could potentially explain treatment difficulties in this phenotype. Unfortunately, treatment efficiency has not been researched in these studies.

OSA patients can be classified using other criteria as well. Due to the small sample size of this study, comorbidities have not been taken into phenotype analysis. However, there is another study which researched stroke patient OSA phenotypes and showed their heterogeneity [15]. According to symptoms and age, 6 phenotypes were identified in a large study, which included more than 18,000 patients in the USA [28]. However, in our study there has not been found a statistically significant difference between age groups. For phenotyping it is not obligatory to use high numbers of various measurements. This has been shown in a study performed in Romania, where phenotypes have been identified by using only 6 essential measurements: age, sex, BMI, blood pressure, neck circumference and Epworth scores [29].

Although there are several studies researching the topic of OSA phenotypes, only two recent studies [13,14] listed above compared treatment effects among different patient groups. Other studies focused more on signifying the heterogeneity of the phenotypes and measuring associating risks, some compared disease outcomes. Hence by investigating the treatment response among phenotypes, we are gradually stepping closer to the discovery of clinically significant patient groups. Thus, more studies comparing treatment effectiveness between phenotypes need to be conducted.

3 phenotypes, described in our study could potentially be easily identified by measuring this data: AHI, PLMS, supine AHI, and BMI. However, more studies need to be conducted, to determine the exact limits of each measurement. These strong differences between phenotypes may indicate different underlying pathophysiological mechanisms. Phenotyping could assist in dividing heterogeneous OSA patients into smaller groups and in choosing appropriate treatment methods which is currently impossible with only measuring AHI rates. However, the clinical significance of these phenotypes, importance to disease outcomes is still not clear and requires further studies.

The main limitation of this study is a small sample size. Another aspect that could have benefited this research is inclusion on data regarding sleeping stages, which was omitted due to data inconsistencies in the database. An inclusion of comorbidities into phenotype analysis, could have improved this study as well. In addition, individual features and their contribution to disease outcomes, response to treatment, adherence to treatment, have not been analyzed in this study. Thus, more comprehensive research is needed to be conducted on phenotype pathophysiology, clinical significance, treatment effectiveness and additional treatment methods in order to contribute to the search of personalized treatment possibilities.

## 5. Conclusions

OSA phenotypes vary significantly when comparing certain anthropometric, clinical and polysomnographic findings and such differences could not be identified by only measuring AHI rates. This phenotype heterogeneity leads to differences in response to treatment with CPAP. Thus, OSA treatment effectiveness depends on OSA phenotypes and treatment techniques, other than CPAP, may be necessary to reach optimal treatment effect. These findings emphasize the importance of a more individualized approach when treating OSA. Future investigations of differences in OSA phenotype treatment effectiveness are needed.

## Figures and Tables

**Table 1:** Characteristics of the study population.

Data	Mean ± SD or Median (IQR)	Minimum-Maximum
Age, years	55.6 ± 10.4	30.0–84.0
Sex:		
Male, %	75.6	
Female, %	24.4	
Height, cm	174.3 ± 9.4	149.0–198.0
Weight, kg	118.2 ± 24.3	68.0–209.0
BMI, kg/m^2^	39.0 ± 7.8	23.5–63.1
Neck circumference, cm	45.2 ± 5.1	31.0–63.0
Waist circumference, cm	126.3 ± 15.5	87.0–178.0
Neck to waist ratio	0.4 ± 0.1	0.3–0.1
Current smoking, %	24.5	
Systolic BP, mmHg	136.1 ± 15.3	98.0–190.0
Diastolic BP, mmHg	83.1 ± 9.9	60.0–120.0
ESS	10.4 ± 5.2	1.0–24.0
EDS, %	76.8	
pH	7.4 ± 0.1	7.3–7.5
SaO_2_, %	95.5 (93.7–96.6)	66.0–100.0
PaCO_2_, mmHg	42.2 (39.6–44.9)	27.5–68.5
PaO_2_, mmHg	72.5 (66.6–80.7)	40.9–146.0
HCO_3_	26.4 ± 2.5	21.0–36.0

Abbreviations: SD–standard deviation, IQR–interquartile range, BMI–body mass index, Current smoking–the section of the study population who were smoking at the time, BP–blood pressure, ESS–Epworth sleepiness scale score, EDS–percentage of subjects experiencing excessive daytime sleepiness, SaO_2_–daytime saturation of oxygen in the blood, PaCO_2_–daytime carbon dioxide levels in the blood, PaO_2_–daytime oxygen levels in the blood, HCO_3_–bicarbonate levels in the blood.

**Table 2:** Polysomnographic data before treatment.

Data	Mean ± SD or Median (IQR)	Minimum-Maximum
AHI, times/hour	50.0 ± 22.9	9.8–112.2
ARI, times/hour	66.1 ± 51.6	0.5–173.2
Mean SpO_2_, %	91.0 (87.6–92.1)	65.0–96.0
Nadir SpO_2_, %	69.0 ± 2.0	27.0–90.0
ODI, times/hour	42.3 ± 24.4	0.6–110.9
Snoring percentage, %	34.4 ± 19.6	1.6–87.3
PLMS, times/hour	28.7 (8.18–67.9)	0.0–212.8
Sleep efficiency, %	81.4 (66.7–93.9)	15.7–99.9
Mean respiratory rate, times/hour	20.1 ± 4.7	11.4–39.6
Supine AHI, times/hour	56.6 ± 25.3	0.0–122.0
Non-supine AHI, times/hour	41.6 ± 28.0	0.0–118.1

Abbreviations: SD–standard deviation, IQR–interquartile range, AHI–apnea-hypopnea index, SpO2–saturation of oxygen in the blood, ARI–arousal index, ODI–oxygen desaturation index, PLMS–periodic limb movement index.

**Table 3:** Clusters and their characteristics.

Data	Positional OSA	Severe OSA	OSA and PLM	*p* Value
Age, years	56.1 ± 10.1	55.3 ± 9.6	56.8 ± 9.3	0.784
BMI, kg/m^2^	37.6 ± 8.0	42.1 ± 6.7	38.3 ± 7.2	0.001
Neck circumference, cm	44.5 ± 5.0	47.7 ± 4.4	44.1 ± 4.6	<0.001
Waist circumference, cm	122.8 ± 14.9	133.9 ± 14.2	127.5 ± 14.5	<0.001
Neck-waist ratio	0.4 ± 0.1	0.4 ± 0.1	0.4 ± 0.1	0.102
Current smoking, %	14.6	28.3	44.0	0.041
Systolic BP, mmHg	135.3 ± 16.6	138.9 ± 12.4	130.3 ± 12.4	0.039
ESS	10.2 ± 5.2	11.2 ± 4.5	8.8 ± 5.2	0.197
EDS, %	71.7	88.1	72.4	0.047
SaO_2_, %	95.9 (94.6–97.1)	95.3 (93.0–96.4)	95.6 (93.3–96.6)	0.090
PaCO_2_, mmHg	41.4 (39.3–44.0)	43.6 (40.8–47.6)	41.8 (38.6–42.5)	0.010
PaO_2_, mmHg	76.0 (68.9–83.7)	68.9 (64.3–78.4)	72.2 (65.0–80.4)	0.024
AHI, times/hour	37.2 ± 15.9	74.0 ± 12.9	45.2 ± 21.2	<0.001
ARI, times/hour	62.3 ± 52.9	63.6 ± 47.2	92.3 ± 52.1	0.021
Mean SpO_2_, %	92.0 (90.5–93.0)	88.1 (84.8–90.8)	91.0 (87.3–92.0)	<0.001
Nadir SpO_2_, %	73.0 ± 10.0	63.1 ± 10.2	72.7 ± 8.4	<0.001
ODI, times/hour	31.2 ± 17.9	63.2 ± 22.6	41.1 ± 25.3	<0.001
Snoring percentage, %	35.0 ± 20.9	26.4 ± 15.0	42.4 ± 17.9	0.007
PLMS, times/hour	16.5 (5.5–46.6)	34.4 (13.9–52.2)	124.9 (105.2–164.1)	<0.001
Sleep efficiency, %	81.2 (66.0–94.2)	80.4 (63.9–92.7)	90.2 (68.5–97.8)	0.407
Supine AHI, times/hour	47.9 ± 21.9	76.4 ± 19.6	47.5 ± 26.9	<0.001
Non supine AHI, times/hour	24.8 ± 17.2	71.4 ± 17.2	39.4 ± 26.1	<0.001

Results are expressed by mean ± standard deviation and median (interquartile range). Abbreviations: BMI–body mass index, Current smoking–the section of the study population who were smoking at the time, BP–blood pressure, ESS–Epworth sleepiness scale score, EDS–percentage of subjects experiencing excessive daytime sleepiness, SaO_2_–daytime saturation of oxygen in the blood, PaCO_2_–daytime carbon dioxide levels in the blood, PaO_2_–daytime oxygen levels in the blood. AHI–apnea-hypopnea index, Mean SpO_2_–average saturation of oxygen in the blood during polysomnography, Nadir SpO_2_–lowest saturation of oxygen in the blood during polysomnography, ARI–arousal index, ODI–oxygen desaturation index, PLMS–periodic limb movement index.

## Data Availability

The original datasets are not publicly available due to data protection policies. The data presented in this study are available on scientific request from the corresponding author.
